# Optimizing Timing and Dose of Starting Norepinephrine and Vasopressin in Septic Shock

**DOI:** 10.3390/life16060913

**Published:** 2026-05-29

**Authors:** Gaku Hiroto, Mitsuaki Nishikimi, Nobuaki Shime

**Affiliations:** 1 Department of Emergency and Critical Care Medicine, Graduate School of Biomedical and Health Sciences, Hiroshima University, Hiroshima 734-8551, Japan; gh0404@icloud.com (G.H.);; 2 Department of Emergency and Critical Care Medicine, Aichi Medical University, Nagakute 480-1195, Japan

**Keywords:** vasopressor, shock, hypotension, arterial pressure, sepsis

## Abstract

Despite advances in septic shock management, optimal vasopressor strategies remain understudied. Norepinephrine (NE) is recommended as the first-line vasopressor for restoring arterial pressure; however, excessive catecholamine exposure has been associated with adverse events, including arrhythmias, ischemia, and poor clinical outcomes. While the early initiation of NE is increasingly recognized as important, uncertainty persists regarding the optimal starting dose and escalation strategy. In septic shock, particularly refractory septic shock, reduced vascular responsiveness may limit the effectiveness of escalating NE doses and increase the risk of dose-related complications. Vasopressin (AVP), a non-adrenergic vasopressor, provides complementary mechanisms to NE and may reduce catecholamine requirements. Randomized trials have not consistently demonstrated a survival benefit; AVP may improve hemodynamic stability and renal perfusion. Emerging evidence suggests the potential advantages of earlier AVP initiation at lower NE doses than those currently recommended. Collectively, the current evidence supports a strategy that prioritizes early and adequately dosed NE to achieve rapid hemodynamic stabilization, followed by the timely initiation of AVP once moderate NE requirements are reached, rather than the continued escalation of NE alone. Such an integrated approach may help balance efficacy and safety, and minimize catecholamine-related harm while optimizing perfusion in septic shock cases.

## 1. Introduction

Septic shock is characterized by arterial hypotension and tissue hypoperfusion caused by profound vasodilation and vascular hyporesponsiveness [1]. Initial resuscitation includes rapid fluid administration to restore venous return and improve cardiac output; however, only approximately 50% of patients are fluid responsive, and excessive fluid loading may lead to harm [2]. Therefore, the rapid administration of vasopressors is a reasonable option for early resuscitation. The 2026 Surviving Sepsis Campaign Guidelines (SSCG 2026) recommend maintaining a mean arterial pressure (MAP) ≥65 mmHg using vasopressors in combination with initial fluid resuscitation [2].

Norepinephrine (NE) is the first-line vasopressor for patients with septic shock. However, NE administration carries the risk of excessive catecholamine exposure, which is associated with several harmful effects, including arrhythmias, ischemic complications, and worse outcomes [2,3]. In cases where the MAP remains low despite NE administration, such as refractory septic shock, the use of second-line vasopressors, such as vasopressin (AVP), is considered. AVP is a non-adrenergic vasopressor that can restore vascular tone through non-adrenergic pathways and reduce catecholamine requirements in patients with septic shock [4]. Although NE and AVP are widely used in clinical practice, their optimal initial dose and timing of initiation remain unclear. While AVP is often reserved for refractory shock after NE escalation, emerging evidence suggests the potential benefits of earlier adjunctive use [5].

This review summarizes the current evidence regarding the pharmacological rationale, timing, and dosing of NE in septic shock and discusses the physiological and clinical considerations for adjunctive AVP therapy. A practical strategy is proposed, emphasizing early and sufficiently dosed NE followed by timely AVP initiation.

## 2. Methodology

This narrative review synthesized the current evidence regarding the timing of vasopressor initiation and initial dosing strategies in patients with septic shock, with particular emphasis on norepinephrine and vasopressin, as well as their effects on hemodynamic stabilization, organ perfusion, and clinical outcomes. To identify the relevant literature, an extensive search was conducted focusing on the core concepts of the review topic and related physiological mechanisms. In accordance with standard approaches for narrative scientific reviews, the biomedical database PubMed was searched using combinations of relevant keywords integrated with Boolean operators (“AND” and “OR”). Only articles published in English and available up to March 2026 were included in this review. The search strategy included combinations of the following terms:

Pharmacological Agents: “Norepinephrine,” “Vasopressin,” “Angiotensin II,” and “vasopressor.”

Clinical Conditions: “septic shock,” “vasodilatory shock,” “distributive shock,” and “refractory septic shock.”

Vasopressor Strategies: “vasopressor dose,” “vasopressor timing,” “vasopressor escalation,” “peripheral vasopressor administration,” and “non-adrenergic vasopressor.”

Complications: “ischemic complications,” and “vasopressor-related adverse effects.”

## 3. Norepinephrine in Septic Shock

### 3.1. Pharmacological Rationale

NE is a catecholamine that possesses both potent α_1_-adrenergic receptor-mediated vasoconstriction and β_1_-adrenergic receptor-mediated positive inotropic effects [6]. NE predominantly stimulates α_1_-adrenergic receptors, produces arterial and venous vasoconstriction, thereby increasing systemic vascular resistance and arterial pressure while also augmenting stressed intravascular volume and cardiac preload, ultimately improving organ perfusion in distributive shock such as septic shock [7]. Furthermore, NE exerts modest β_1_-adrenergic stimulation that enhances myocardial contractility and may increase cardiac output despite its predominant vasoconstrictor profile [8]. This β_1_-mediated inotropic effect may partially counteract sepsis-induced myocardial depression and help maintain cardiac output in septic shock [9].

Several guidelines recommend NE as the first-line vasopressor for septic shock [2,10], which is supported by extensive evidence accumulated from randomized controlled trials (RCTs) and meta-analyses comparing it with other catecholamines. A few RCTs comparing the beneficial effect of dopamine with NE have established an initial evidence base for their relative efficacy in septic shock. Martin et al. demonstrated that NE achieved a more consistent restoration of MAP and was associated with fewer arrhythmias than dopamine in patients with hyperdynamic septic shock characterized by a cardiac index >4.0 L/min/m^2^ [11]. Subsequently, Patel et al. conducted a larger randomized trial demonstrating comparable hemodynamic efficacy between the two groups but a higher incidence of arrhythmias in the dopamine group for patients with septic shock [12]. Their findings were later confirmed even in patients with cardiogenic shock, in the large multicenter SOAP II trial, in which dopamine was associated with significantly more arrhythmic events and worse outcomes than NE [13]. A subsequent meta-analysis by De Backer et al. demonstrated lower mortality and fewer arrhythmic complications with NE, supporting its role as the preferred first-line vasopressor for septic shock [14]. Regarding the comparison of NE with other vasopressors, including epinephrine and AVP, the superiority of NE has not been completely established [15,16]; however, findings from multiple systematic reviews and meta-analyses have consistently indicated that NE has a more favorable safety profile, thereby supporting its preferential use as the first-line vasopressor in septic shock [17,18].

### 3.2. Optimal Timing of Norepinephrine Initiation

The appropriate timing of NE initiation in septic shock has not yet been fully established. Previous observational studies have suggested that prolonged hypotension is associated with increased mortality [19]. Therefore, it is reasonable to consider that the rapid restoration of perfusion can improve outcomes, and its achievement through the early initiation of NE has been increasingly recognized as a key component of resuscitation in septic shock [20]. The early use of NE can also help reduce the amount of fluid administered by augmenting the stressed volume and cardiac preload [7]. A previous RCT, the Control of Early Norepinephrine in Septic Shock Resuscitation (CENSER) trial, demonstrated that early low-dose NE significantly shortened the time to achieve the target MAP and reduced cardiopulmonary complications compared with delayed initiation [21]. Recently, meta-analytic evidence integrating randomized and observational data further confirmed the associations between early NE use and decreased mortality, including reduced fluid overload [22]. Consistent with this evidence, SSCG 2026 recommends the initiation of NE within the first hour of resuscitation when hypotension persists in septic shock [2]. Future studies are warranted to determine the optimal initial dosing and escalation strategies for NE during the early phases of septic shock resuscitation.

### 3.3. Negative Impacts of Excessive Exposure to Norepinephrine

Septic shock is characterized by a decreased vascular responsiveness to adrenergic agents [23], and as a result, in the most severe cases, common doses of NE are sometimes insufficient to correct hypotension. In these situations, excessively high-dose NE has been used to treat septic shock, although several observational studies have consistently shown that it is associated with poor clinical outcomes. Brown et al. reported outcomes in patients with shock requiring high-dose vasopressor therapy (≥1.0 μg/kg/min NE equivalent), which was only 17% survival [24]. In this context, a multicenter observational study by Dünser et al. demonstrated that an increased vasopressor load was independently associated with mortality due to septic shock [25]. However, it should be noted that these findings do not necessarily indicate that high-dose vasopressors directly cause mortality, but rather suggest that they may represent a marker of extremely severe shock.

High-dose NE has been associated with cardiovascular complications, including atrial fibrillation and ischemic events [26]. In an observational study of 250 patients with septic shock, dysrhythmias occurred in 34% of the patients and were associated with significantly higher mortality and fewer intensive care unit (ICU)-free and ventilator-free days. Notably, both the duration of NE infusion and maximum dose were independently associated with an increased risk of dysrhythmia, suggesting a dose-dependent relationship between NE exposure and arrhythmic risk [27]. Furthermore, high-dose NE may cause severe peripheral vasoconstriction, leading to limb ischemia and tissue necrosis, necessitating surgical interventions such as debridement or amputation in approximately 50% of patients, particularly those with shock requiring multiple vasopressors [28]. Although no clear NE dose threshold has been established for these adverse effects, these findings highlight the potential harm caused by escalating NE doses.

**Figure 1:**
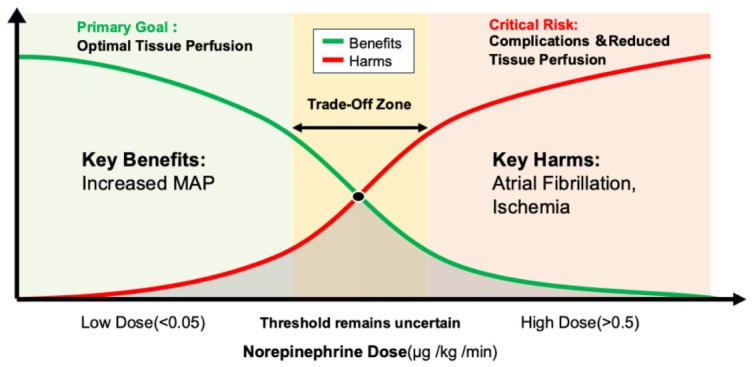
Norepinephrine trade-off concept.

Although a direct causal relationship has not been clearly established, it may be partially explained by the reduced vascular responsiveness observed in the pathophysiological condition of septic shock. Levy et al. reviewed the mechanisms of vascular hyporesponsiveness to vasopressors in septic shock, by which the release of inflammatory mediators, overproduction of nitric oxide, and alterations in adrenergic receptor signaling contribute to vasoplegia [23]. Interestingly, this reduced responsiveness has been confirmed in a recent observational clinical study. Bosch et al. reported that the arterial pressure response to NE diminishes as the baseline NE dose increases, with a markedly attenuated MAP response observed at doses ≥0.3 μg/kg/min in patients with septic shock [29]. Recently, refractory septic shock has been defined as persistent hyperlactatemia and/or prolonged capillary refill time (CRT) in a patient with septic shock who is unresponsive to fluid resuscitation and requires a NE equivalent dose >0.5 μg/kg/min, with mixed shock excluded by critical care ultrasound [30]. Although the 0.5 μg/kg/min threshold was determined by expert consensus, impaired vascular responsiveness to vasopressors may begin at lower doses, at approximately 0.3 μg/kg/min, in the pathophysiology of refractory septic shock. These findings suggest that increasing NE doses in patients with refractory septic shock may diminish hemodynamic improvements and potentially increase the risk of dose-related adverse effects. Because the effect of NE infusion involves a trade-off, an excessive NE dose may be harmful rather than beneficial for patients with refractory septic shock (Figure 1). Further studies focusing on excessive NE exposure are required.

### 3.4. Optimal Initial Dose of Norepinephrine

There is no universally established standard starting dose of NE in patients with septic shock. Many clinical trials generally initiate NE at approximately 0.05–0.1 μg/kg/min, with subsequent titration to achieve the target MAP, typically ≥65 mmHg [2]. Recently, the present group successfully visualized early dynamic MAP trajectories before and after NE initiation using a large ICU database with high-resolution time-series vital signs. This study showed that higher initial NE doses (0.1 μg/kg/min) were associated with both larger and faster MAP increases, and lower starting doses (0.025 or 0.05 μg/kg/min) appeared insufficient to achieve a target MAP within 60 min in patients with severe hypotension. Also, the speed of MAP increase in patients with sepsis receiving a very low dose of NE was lower compared to patients without sepsis receiving the same dose, suggesting sepsis-specific mechanism impairing vasopressor responsiveness [31].

Clinicians may hesitate to initiate NE at higher doses because of concerns regarding adverse events, including peripheral ischemia and tissue necrosis, particularly when administered through peripheral venous access. However, previous studies have supported the safety of peripheral NE administration at doses of 0.1–0.2 μg/kg/min [32,33]. Altogether, initiating NE at overly conservative doses, such as <0.1 μg/kg/min, may delay the restoration of adequate perfusion pressure, particularly in patients with severe hypotension. Therefore, an initial dose of approximately 0.1 μg/kg/min or higher may represent a reasonable strategy in selected patients with septic shock, although the optimal starting dose should be individualized based on the severity of hypotension and the patient’s underlying cardiovascular conditions. Patients with severe hypotension may require more rapid dose escalation, whereas those with arrhythmias, ischemic heart disease, peripheral vascular disease, or low cardiac output may require more cautious titration. Further prospective studies are needed to determine the optimal initial dosing strategy for different patient subgroups.

Careful monitoring remains essential during peripheral NE administration. Frequent assessment of the infusion site for pain, swelling, blanching, or impaired distal perfusion is important for the early detection of extravasation or local ischemic complications. In addition, continuous monitoring of heart rate, cardiac rhythm, peripheral perfusion, urine output, lactate concentration, and CRT is recommended, particularly in patients receiving higher NE doses or those with underlying arrhythmias, ischemic heart disease, or peripheral vascular disease [2].

## 4. Vasopressin in Septic Shock

### 4.1. Mechanistic Rationale

AVP is an endogenous non-catecholamine vasopressor secreted by the posterior pituitary gland. AVP has numerous functions in the body, including promotion of water reabsorption via V2 receptors in the renal collecting ducts and modulation of stress hormone responses via V1b receptors in the pituitary gland [34]. One of the most well-known functions of AVP is vasoconstriction through vascular smooth muscle V1a receptors, and the fact that this mechanism is independent of NE is clinically important [35]. In patients with septic shock, circulating AVP levels are inappropriately low despite ongoing hypotension, indicating a relative AVP deficiency that may contribute to persistent vasodilation [36]. Sharshar et al. further demonstrated that AVP secretion follows a biphasic pattern, with an initial transient increase during the early phase followed by a decline associated with impaired neuroendocrine regulation when hemodynamic stability is not restored within 24–48 h [37]. In their article, the authors suggested that this might be due to the depletion of endogenous AVP stores and dysregulation of the hypothalamic–pituitary axis. Although the mechanism underlying decreased circulating AVP levels in patients with septic shock is unclear, these pathophysiological features provide a mechanistic rationale for exogenous AVP supplementation.

Unlike NE, AVP exerts beneficial effects on renal hemodynamics. It preferentially constricts the efferent arterioles via V1a receptor activation, while having a minimal effect on afferent arterioles, possibly through local nitric oxide-mediated vasodilation. This combination may increase intraglomerular hydrostatic pressure and help maintain the glomerular filtration rate (GFR) [38]. Consistent with this physiological effect, AVP has been associated with increased urine output and creatinine clearance compared to catecholamines alone. The Vasopressin and Septic Shock Trial (VASST) and the Vasopressin vs. Norepinephrine as Initial Therapy in Septic Shock (VANISH) trial did not consistently demonstrate significant improvements in renal outcomes; however, the findings of the VANISH trial suggested that AVP administration may reduce the need for renal replacement therapy (RRT) [5,16]. In a meta-analysis by Kotani et al., including 51 RCTs, the use of non-adrenergic vasopressors was associated with a reduced risk of RRT, particularly in patients at risk of acute kidney injury, based on a subset of four trials reporting RRT outcomes [39].

Despite these physiological advantages, randomized trials have not consistently demonstrated survival benefits of AVP. The VASST showed that low-dose AVP (0.01–0.03 U/min) did not reduce 28-day mortality compared with NE in the overall population. However, a potential benefit was observed in patients with less-severe shock, whereas the VANISH trial found no significant difference in mortality between early AVP and NE strategies [5,16]. These findings are supported by an individual patient data meta-analysis by Nagendran et al., which showed no overall survival benefit, although a signal toward improved 28-day survival at lower vasopressor doses was not sustained in the long-term outcomes [40]. Collectively, the current evidence does not support a consistent improvement in patient-centered outcomes with AVP. Despite the lack of a consistent survival benefit, the administration of AVP as an adjunct to NE may be a rational strategy for enhancing hemodynamic stability, reducing catecholamine requirements, and preserving renal perfusion.

### 4.2. Optimal Timing of Vasopressin Initiation

Current guidelines recommend initiating AVP when NE requirements reach approximately 0.25–0.50 μg/kg/min [2,10]. However, in the VASST, AVP was associated with a survival signal in patients receiving lower baseline NE doses (<15 μg/min) [5]. In the VANISH trial, AVP was initiated relatively early during shock management at a median NE dose of approximately 0.16 μg/kg/min (interquartile range [IQR] 0.10–0.31), and was associated with reduced RRT requirements when incorporated early into vasopressor escalation strategies [16]. Furthermore, a recent meta-analysis defining early AVP initiation as within 6 h of septic shock onset demonstrated improved hemodynamic stability and shorter hospital stay compared to delayed (>6 h) or no AVP use [41]. Collectively, these findings support the potential benefits of earlier AVP initiation in terms of both lower NE thresholds and earlier timing after shock onset.

The optimal timing for AVP initiation remains uncertain; however, emerging data suggest that AVP may be introduced at lower NE doses than currently recommended. The Optimal Vasopressin Initiation in Septic Shock (OVISS) reinforcement learning study indicated that optimal strategies favored AVP initiation at a median NE dose of approximately 0.20 μg/kg/min, substantially lower than the 0.37 μg/kg/min observed in clinical practice, aiming to achieve early MAP recovery before severe vasoplegia becomes fully established [42]. Altogether, these findings suggest that earlier AVP initiation at NE doses of approximately 0.2 μg/kg/min may represent a physiologically rational and promising strategy. However, this approach remains investigational and should not be interpreted as a guideline-based recommendation. Future studies evaluating individualized AVP strategies based on endogenous vasopressin levels or related biomarkers may further help optimize patient selection and timing of AVP initiation.

### 4.3. Optimal Dosing of Starting Vasopressin

In most clinical trials, AVP has been administered at a fixed dose of 0.03 U/min, a regimen adopted in major randomized studies such as VASST and VANISH [5,16]. Unlike catecholamines, AVP is not typically titrated to blood pressure targets, reflecting its pharmacological profile, including early V1 receptor engagement and a relatively flat dose–response relationship within the clinically used range [43]. However, the clinical evidence regarding the optimal AVP dose remains limited. It is therefore possible that a fixed dose of 0.03 U/min may be insufficient in some patients, similar to concerns regarding the initial dosing of NE discussed above. Further studies are warranted to determine the optimal AVP dosing strategy.

Adverse effects of AVP appear to be dose-dependent and are primarily related to excessive vasoconstriction. At physiologic doses, AVP (typically 0.01–0.04 U/min) is generally considered relatively safe and is commonly administered without routine dose titration. In contrast, higher doses of AVP (>0.04 U/min) have been associated with impaired splanchnic and peripheral perfusion, potentially increasing the risk of ischemic complications [38]. Clinically, ischemic complications may manifest as peripheral or digital ischemia with cyanosis or necrosis of the extremities, while mesenteric or myocardial ischemia can occur in severe cases [40,44]. Ischemic skin lesions have also been reported, particularly in patients with obesity [44]. Therefore, careful monitoring for signs of ischemic complications remains important, particularly in patients receiving higher AVP doses.

## 5. Alternative Vasopressor Option

In patients with septic shock who remain hypotensive despite adequate fluid resuscitation and high-dose catecholamines, angiotensin II has emerged as a potential adjunctive vasopressor targeting the renin–angiotensin–aldosterone system. As the final effector peptide of this system, angiotensin II exerts potent vasoconstrictive effects via angiotensin II type 1 (AT1) receptors through a non-adrenergic pathway, leading to a rapid increase in systemic vascular resistance and blood pressure [45]. The Angiotensin II for the Treatment of High-Output Shock 3 (ATHOS-3) trial demonstrated that angiotensin II significantly increased MAP compared with placebo, confirming its potent vasoconstrictive effect in catecholamine-refractory vasodilatory shock (defined as persistent hypotension despite adequate fluid resuscitation and NE equivalent dose >0.2 μg/kg/min for 6–48 h in patients with preserved cardiac output), although it did not show a statistically significant reduction in mortality [45]. Nevertheless, subsequent post hoc analyses of ATHOS-3 suggested potential renal benefits. In a subgroup of patients requiring renal replacement therapy (RRT), angiotensin II was associated with higher rates of liberation from RRT and improved survival [46], whereas another analysis including a broader population of patients with acute kidney injury demonstrated a reduction in mortality and increased RRT-free days specifically among patients with stage 3 acute kidney injury [47]. Subsequent systematic reviews and meta-analyses of non-adrenergic vasopressors, including angiotensin II, suggest a possible reduction in mortality. Safety outcomes—including ventricular arrhythmias, ischemic complications, and thromboembolic events—remain uncertain, with very low-certainty evidence [40]. The SSCG 2026 classifies the evidence for angiotensin II as very low certainty, supporting its use only as a rescue or adjunctive vasopressor in refractory septic shock, with uncertain effects on mortality and other patient-centered outcomes [2]. The major clinical trials and meta-analyses evaluating vasopressor strategies in septic shock are summarized in Table 1.

## 6. Conclusions

**Figure 2:**
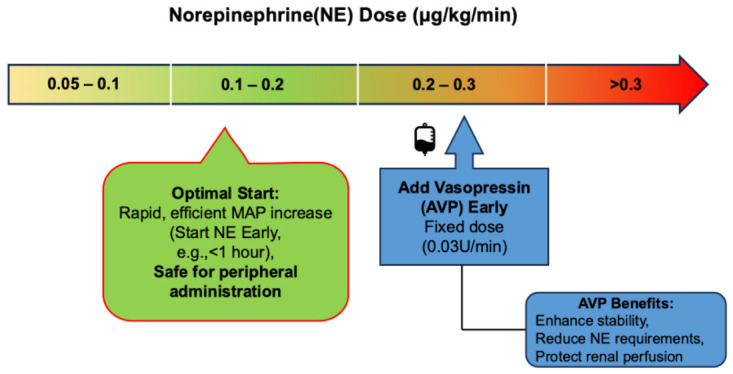
Integrated vasopressor strategy in septic shock optimizing norepinephrine and vasopressin. NE is initiated early after fluid resuscitation at a sufficient starting dose (≥0.1 μg/kg/min) to achieve a target MAP (MAP ≥65 mmHg). Hemodynamic response should be reassessed within 30–60 min using both MAP and markers of tissue perfusion. In patients with an inadequate response or escalating norepinephrine requirements, early addition of vasopressin may be considered when the norepinephrine dose reaches approximately 0.2 μg/kg/min.

NE should remain the first-line vasopressor in septic shock and should be initiated early to achieve rapid hemodynamic stabilization. Initiation at approximately 0.1 μg/kg/min or higher may be a physiologically rational strategy supported by emerging evidence. Persistent dose escalation may indicate catecholamine refractoriness rather than insufficient therapy. In such cases, relatively early addition of AVP may be a reasonable adjunctive strategy. An integrated approach combining adequately dosed early NE with timely AVP initiation may offer advantages over continued NE escalation alone. However, it should be emphasized that all currently available evidence supporting this strategy is derived exclusively from observational studies and post hoc analyses, without confirmation from randomized controlled trials. Therefore, further prospective studies are warranted to confirm its efficacy and safety (Figure 2).

## Figures and Tables

**Table 1:** Major clinical trials and meta-analyses evaluating vasopressor strategies in septic shock.

	CENSER (2019) [21]	Shi et al. (2025) [22]	VASST (2008) [5]	VANISH (2016) [16]	Nagendran et al. (2019) [40]	Mamede et al. (2026) [41]	OVISS (2025) [42]	ATHOS-3 (2017) [45]	Kotani et al. (2024) [39]
**Design**	Single-center RCT	Systematic review and meta-analysis	Multicenter double-blind RCT	Multicenter double-blind factorial RCT	Individual patient data meta-analysis	Systematic review and meta-analysis	Reinforcement learning analysis	Multicenter double-blind RCT	Systematic review and meta-analysis
**Population**	Septic shock requiring vasopressors after fluid resuscitation (*n* = 310)	Septic shock (10 studies; *n* = 4767)	Septic shock requiring catecholamine vasopressors (*n* = 778)	Septic shock requiring vasopressors despite fluid resuscitation (*n* = 409)	Septic shock (4 RCTs; *n* = 1453)	Septic shock (6 studies; *n* = 1167)	Multicenter septic shock cohorts receiving norepinephrine (*n* = 14,453)	Catecholamine-resistant vasodilatory shock (*n* = 321)	Vasodilatory shock and perioperative vasoplegia (51 RCTs; *n* = 5715)
**Intervention**	Early fixed-dose NE (0.05 μg/kg/min) vs. placebo-controlled standard care	Early vs. delayed norepinephrine initiation	Low-dose AVP (0.01–0.03 U/min) vs. norepinephrine	Early AVP (up to 0.06 U/min) vs. norepinephrine	AVP therapy vs. other vasoactive strategies	Early AVP plus NE vs. NE alone or delayed AVP	Reinforcement learning-guided early AVP initiation	Angiotensin II vs. placebo	Non-adrenergic vasopressors vs. adrenergic vasopressors alone or placebo
**Timing strategy**	During initial resuscitation	Particularly within 1 h	At NE requirement ≥5 μg/min	Within 6 h of shock onset	Variable across included RCTs	Early vs. delayed/no AVP initiation	At lower NE dose (~0.2 μg/kg/min)	At NE equivalent dose >0.2 μg/kg/min	Variable across included RCTs
**Main findings**	Early NE improved shock control and accelerated MAP recovery	Early NE associated with lower mortality, less fluid administration, and faster MAP achievement	AVP did not reduce overall mortality but showed possible benefit in less severe shock	Early AVP did not improve kidney failure–free days but reduced RRT use	AVP did not reduce mortality but may reduce RRT use and arrhythmias	Early AVP associated with shorter hospital stay without clear mortality benefit	Earlier AVP initiation aligned with reinforcement learning models was associated with lower in-hospital mortality.	Angiotensin II significantly improved MAP in refractory shock	Non-adrenergic vasopressors showed pooled survival benefit signal in septic shock
**Key limitations/interpretation**	Mortality benefit not definitive; single-center study	Significant heterogeneity; further RCTs are needed	Benefit signal limited to subgroup analysis	Reduced RRT use without clear renal outcome benefit	Reduced RRT signal was not robust in sensitivity analyses	Most outcomes unchanged; high risk of bias in included studies	Observational AI-derived analysis requiring prospective validation	Hemodynamic benefit without proven mortality reduction	No individual agent demonstrated consistent survival benefit

## Data Availability

No new data were created or analyzed in this study. Data sharing is not applicable to this article.

## Abbreviations

The following abbreviations are used in this manuscript:

NE	norepinephrine
AVP	vasopressin
MAP	mean arterial pressure
